# Temporal Scientific Signals Associated with Regulatory Milestones in Anatomically Targeted Therapeutics: Evidence from Botulinum Toxin Research

**DOI:** 10.3390/toxins18100424

**Published:** 2026-10-01

**Authors:** Yue Lin, Yiseul Choi, Xiaoqing Liu, Hee-Dae Jeon, Hyun Jin Park

**Affiliations:** 1 Department of Advanced General Dentistry, Yonsei University College of Dentistry, Seoul 03722, Republic of Korea; limgup@yuhs.ac (Y.L.); loa@yuhs.ac (Y.C.); 2 Yonsei Institute for Digital Health, Yonsei University, Seoul 03722, Republic of Korea; 3 Department of Radiation Oncology, Cancer Hospital of Shantou University Medical College, Shantou 515000, China; liuxiaoqing.stu@gmail.com; 4 Banobagi Dermatology Clinic, Seoul 06109, Republic of Korea; jeonskin@naver.com; 5 Department of Anatomy, College of Medicine, Konkuk University, Chungju 27478, Republic of Korea

**Keywords:** botulinum neurotoxin, scientometrics, translational lag, regulatory approval, anatomy-guided interventions

## Abstract

Regulatory approval may occur before or after a research topic becomes prominent in the scientific literature. We examined this temporal relationship using botulinum neurotoxin (BoNT) as a longitudinal case study. We analyzed 2065 anatomy-related BoNT publications indexed in Web of Science and Scopus from 1983 to 2024. The literature was divided into 16 periods based on U.S. FDA approval dates for onabotulinumtoxinA, and indication-related keyword rankings and thematic clusters were compared with the corresponding approval dates. Among the 10 indication groups examined, 7 showed prominent indication-related keywords before FDA approval, 2 showed signals around the time of approval, and 1 showed a post-approval signal. We describe these patterns as “proactive exploration” when scientific attention became prominent before approval and “reactive catalysis” when it became more prominent around or after approval. Differences in the observed lag may be related to target anatomical complexity and market orientation, but this remains conceptual. Overall, the findings show that time-resolved scientometrics can retrospectively identify scientific patterns associated with regulatory milestones. The approach is hypothesis-generating and requires prospective validation before it can be used for regulatory forecasting.

## 1. Introduction

Regulatory approvals represent critical inflection points in technological innovation systems. In biomedicine, decisions by regulatory authorities such as the United States Food and Drug Administration (FDA) do not merely authorize market entry; they reshape standards of care, redirect research priorities, and legitimize new therapeutic paradigms [1,2]. Yet, despite the central role of these milestones, the temporal relationship between scientific knowledge accumulation and regulatory transition remains insufficiently clarified. Does scientific attention tend to emerge before regulatory milestones, or does it show up primarily after regulatory validation?

Scientometrics provides a quantitative framework to address this question. Unlike narrative reviews constrained by subjective selection and limited scope, scientometric approaches map the structural evolution of knowledge domains over time [3]. By constructing keyword co-occurrence networks and thematic clusters using tools such as CiteSpace, it becomes possible to trace how research foci emerge, expand, and reorganize across defined temporal segments [4]. When anchored to external regulatory events, such analyses can reveal whether scientific attention precedes, coincides with, or follows approval milestones.

Botulinum neurotoxin (BoNT) offers a particularly suitable longitudinal case for examining these dynamics. Over the past four decades, BoNT has evolved from a biological hazard into a cornerstone of therapeutic and aesthetic medicine [5,6]. The expansion of its clinical indications, from ophthalmologic disorders to spasticity, migraine, urological dysfunction, and cosmetic applications, has been marked by successive FDA approvals. As the first-in-class and long-standing market leader, onabotulinumtoxinA (Botox^®^) provides a coherent and traceable regulatory timeline. In this study, we use the FDA approval history of Botox as a consistent regulatory anchor to segment the broader BoNT research landscape. This strategy does not equate brand with class; rather, it uses one well-documented product history as a consistent temporal reference for examining changes in the broader literature.

Within this evolving field, anatomy-related research occupies a pivotal yet understudied position. Anatomical investigation is intuitively assumed to underpin clinical translation by defining targets, refining delivery techniques, and reducing procedural risk. However, the directionality of this relationship remains unclear. Does anatomy-driven research function as proactive exploration, mapping new indications years before formal approval? Or does it operate as reactive catalysis, intensifying only after regulatory validation to optimize precision and safety? The absence of a systematic, time-resolved analysis has left this translational sequence insufficiently understood. Scientific interest, technical maturation, clinical evidence, commercial adoption, and regulatory approval are related but distinct processes and should not be treated as interchangeable signals.

To address this gap, we apply scientometric methods to anatomy-related BoNT literature indexed in Web of Science and Scopus from 1983 to 2024, organized across 16 FDA approval periods. We intentionally adopt a broad definition of “applied clinical anatomy,” encompassing not only classical cadaveric and morphological studies but also research on injection-site localization, guidance technologies (e.g., ultrasound, EMG), needle trajectory, diffusion control, and safety optimization. In injection-based therapies, anatomical knowledge extends beyond static structure to include spatial accessibility, target depth, and procedural reproducibility [7]. By aligning anatomy-driven research hotspots with regulatory milestones, this study aims to (1) characterize the temporal relationship between scientific attention and FDA approvals; (2) distinguish patterns of proactive exploration and reactive catalysis; (3) explore whether translational lag may be related to structural complexity and non-therapeutic orientation for anatomy-guided interventions; and (4) use this case as a proof-of-concept to examine whether time-aligned scientometric patterns can identify potential scientific signals associated with subsequent regulatory milestones. More broadly, this study explores whether retrospective publication patterns can provide a basis for hypothesis generation regarding regulatory transitions.

## 2. Results

A per-period analysis was conducted based on the FDA approval dates presented in Table 1, encompassing the following 16 periods: 1983.01–1989.12, 1989.12–2000.12, 2000.12–2002.12, 2002.12–2004.07, 2004.07–2010.03, 2010.03–2010.10, 2010.10–2011.08, 2011.08–2013.01, 2013.01–2013.09, 2013.09–2016.01, 2016.01–2017.10, 2017.10–2019.06, 2019.06–2019.10, 2019.10–2020.07, 2020.07–2021.02, and 2021.02–2024.10. We defined our time periods based on key FDA approval milestones rather than arbitrary fixed intervals, such as 5-year blocks. Since drug development research is usually event-driven rather than linear, applying fixed timelines creates artificial boundaries that can obscure genuine trends. By aligning our analysis with these specific “regulatory eras,” we can examine shifts in anatomical focus in relation to FDA decisions rather than through rigid calendar segmentation. Keywords within each period underwent a synonym merging process to consolidate morphological and spelling variants (e.g., singular/plural forms, capitalization, and synonyms). For instance, terms with distinct hierarchical meanings were not merged, such as botulinum toxin (as a family) and botulinum toxin type A/B (as specific serotypes). During data preprocessing, we deliberately preserved taxonomic distinctions between different formulations and serotypes. For example, “botulinum toxin type A” and “botulinum toxin type B” were treated as independent keyword nodes and were not merged. This approach allowed subsequent clustering analyses to objectively identify research hotspots unrelated to Botox (e.g., type B-focused clusters), thereby demonstrating that our method can effectively distinguish field-wide trends from product-specific trajectories rather than conflating the two. In contrast, variants like facial muscles and face muscles were standardized to facial muscles. The resulting top 10 keywords for each period, following this consolidation, are presented in Table 2. In CiteSpace, the g-index was used as the node-selection criterion within each time slice, while keyword occurrence frequency was used to identify the most prominent keywords within each regulatory period. Therefore, the Top-10 ranking represents within-period thematic prominence rather than absolute research intensity across periods.

To make sure the research hotspots from CiteSpace are really comparable to the FDA indication timeline, we further cleaned the CiteSpace keywords and kept only those that can be mapped directly to (1) disease/indication, (2) target, and (3) specific intervention. This represents how FDA labels are structured around indications, active components, and the marketed products.

We removed three groups of terms that do not carry indication or mechanistic signals from the keyword list: (1) demographic tags (e.g., humans, male, female); (2) study-design and publication-type terms (e.g., article, review, clinical trial); and (3) generic procedure and outcome terms (e.g., injections, injection site, drug safety). These tended to describe how the study was done or how the drug was given, not what new indication was being explored. Keeping them would dilute the correlations. We also excluded ‘Botox’ and general ‘Anatomy’ terms (e.g., anatomical, anatomy, muscle, face, facial muscle). Those words were either part of our original search strategy or too general, such as facial muscle, which could appear in multiple FDA-approved indications; leaving them in would affect the ranking and make the list less informative. After this cleaning, we obtained the Top-10 keywords shown in Table 3.

**Figure 1:**
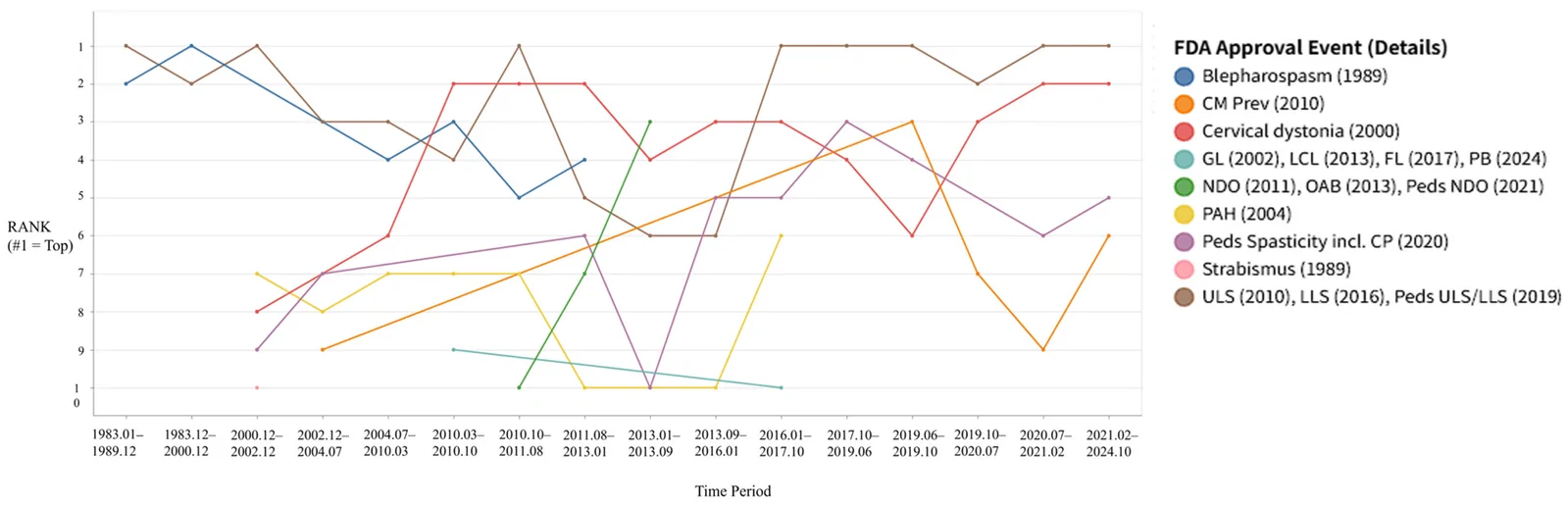
Temporal alignment between FDA approval milestones and the within-period prominence of three representative indication-related keyword groups. Keyword rank is shown for each regulatory period, with a lower rank number indicating greater within-period prominence.

After matching the keywords in Table 3 to FDA-approved indications (Table 4), we plotted the change in their rankings over time, producing the trend shown in Figure 1. Table 2, Table 3 and Table 4 show the successive steps from the original keyword rankings to the cleaned and indication-mapped keywords. Figure 1 then visualizes temporal patterns derived from these rankings. Table 5 provides complementary thematic evidence by showing the major keyword clusters identified within each regulatory period. The figure showed how each FDA-relevant keyword moved in the ranking (with #1 = highest) from 1983 to 2024. It depicted how research key points changed along with the approval periods. Accordingly, changes in rank indicate changes in a keyword’s relative prominence within successive periods, rather than changes in its absolute publication frequency. To ensure visual clarity and simplicity, we selected three representative lines. These lines effectively illustrate two distinct evolutionary modes in the discussion part. To assess whether the indication-keyword mapping influenced the observed temporal patterns, we also performed a sensitivity analysis restricted to direct indication-related terms. Only exact indication terms or close clinical synonyms were retained, while adverse effects, related syndromes, and broader associated terms were excluded. The FDA approval dates and regulatory periods were unchanged.

In this study, a cluster refers to a group of keywords that are more strongly connected to one another within the CiteSpace co-occurrence network, and therefore, represent a shared research theme within a period. The clusters were generated algorithmically by CiteSpace; the cluster numbers (#0, #1, etc.) are identifiers and do not indicate importance or rank. The thematic names shown in Table 5 were assigned by interpreting the representative keywords and top-citing papers within each cluster. After clustering the keywords across the 16 periods, we obtained the corresponding thematic clusters. All of these clusters achieved silhouette scores above 0.79, which indicated that the themes were very coherent internally (keywords within a cluster were highly similar) and clearly distinct from one another. This level of separation supports using these clusters to interpret research trends.

For cluster-label validation, two independent domain experts and the three-member author team performed separate labelling procedures. The author team assigned labels to all 147 clusters based on the representative keywords and top-citing papers. Separately, a stratified sample of 64 clusters (43.53% of all clusters; *n* = 4 from each approval period) was provided to two external domain experts. The experts received only the top keywords and top-citing papers and independently labelled the sampled clusters.

The author team then compared the two expert labels for each of the 64 sampled clusters using a predefined three-point coding system: (1) 2 points (High Agreement): identical or synonymous meaning (e.g., “Cosmetic and Skin-Related Uses” vs. “Aesthetic and Dermatological Applications”); (2) 1 point (Middle Agreement): related theme but different scope (e.g., “Side Effects” vs. “Adverse Events in Chronic Migraine Trials”); and (3) 0 points (No Agreement): completely different topics (e.g., “Chronic Migraine Treatment” vs. “Pediatric Spasticity Management”).

The initial comparison between the two experts yielded 52 High Agreement, 6 Middle Agreement, and 6 No Agreement cases. This corresponded to a Strict Percentage Agreement of 81.25% (52/64) and a Weighted Percentage Agreement of 85.93%, with Middle Agreement counted as 0.5. The two experts then discussed all 64 sampled clusters and reached a consensus label for each cluster. These consensus labels were used as the expert reference set.

Finally, the author team’s independently assigned labels for the same 64 clusters were compared with the expert consensus reference labels using the same three-point criteria. This comparison resulted in 51 High Agreement, 9 Middle Agreement, and 4 No Agreement cases, corresponding to a Strict Percentage Agreement of 79.68% and a Weighted Percentage Agreement of 86.71%. The close agreement between the author-assigned labels and the expert consensus supports the use of the same labelling procedure for the remaining 83 non-sampled clusters [8].

Notably, while we anchored our timeline to Botox approvals, the analysis successfully distinguished independent research trends for other serotypes and formulations. For instance, following the 2000 approval of Myobloc (rimabotulinumtoxin B), ‘botulinum toxin type B’ emerged as a distinct, high-intensity cluster spanning 2002–2010 (Cluster #0). Similarly, a separate cluster for Dysport (abobotulinumtoxinA) appeared in 2016–2017 (Cluster #4), reflecting its specific trajectory in aesthetics.

These findings support the ability of the clustering approach to distinguish research patterns within the broader regulatory timeline.

## 3. Discussion

While our timeline is anchored to Botox, we use this product history as a temporal reference for examining broader changes in anatomy-related BoNT research. For instance, the approval for Chronic Migraine marked an important regulatory milestone, followed by continued knowledge accumulation across other formulations [9]. In this sense, the Botox timeline provides a consistent reference for examining temporal shifts in the broader BoNT literature.

The central finding of this study is a co-evolutionary relationship between the technical knowledge base (conceptualized here as applied clinical anatomy) and regulatory milestones in spatial-dependent pharmacotherapy. Rather than a unidirectional flow from science to market, we identify two distinct modes of knowledge-regulatory interaction. This finding, particularly the evidence for ‘Proactive Exploration’, shows that indication-related scientific attention often became prominent years before subsequent regulatory approval.

The first mode is ‘Proactive Exploration’: technical knowledge accumulation becomes prominent before regulatory approval, with technical questions being explored before formal regulatory validation. This is supported by two key pieces of evidence. First, by comparing the appearance of indication-related keywords with approval milestones, this study revealed that among the 10 indication groups examined in Table 6, 7 (70%) showed a Top-10 indication-related keyword before the corresponding FDA approval, while 2 showed concurrent signals and 1 showed a post-approval signal (Table 6). Specifically, indications characterized by high structural complexity, such as spasticity, cervical dystonia, and hyperhidrosis, emerged as top-ranked keywords on average 4–10 years prior to their respective FDA approvals, with some topics such as spasticity appearing over two decades earlier. Second, our cluster analysis in Table 5 shows that long before the first regulatory milestone for Botox Cosmetic in 2002, ‘Aesthetic and Dermatological Applications’ had already emerged as an independent cluster between 1989.12 and 2000.12. This suggests that technical feasibility was already being explored before later market entry and regulatory adoption. Worth mentioning, “first appearance” in Table 6 refers to the first regulatory period in which at least one keyword mapped to an indication group (Table 4) appeared among the Top-10 cleansed keywords (Table 3). It does not refer to the first publication on that indication or the first occurrence of the term in the full dataset.

The other mode is ‘Reactive Catalysis’, a descriptive pattern in which targeted research activity becomes more prominent around or after FDA approval. The term describes the temporal pattern and does not imply that approval itself caused the increase in research activity. This pattern is clearly visible in aesthetic research: immediately after the 2002 GL approval, several aesthetic themes became prominent in the 2002.12–2004.07 period, evidenced by the emergence of multiple clusters (e.g., #3, #5) focusing on anatomical refinement, spatial precision, and safety optimization. A similar pattern of increased thematic prominence was observed for strabismus and chronic migraine, which also demonstrated a significant post-approval surge in research activity. This post-approval increase may reflect several factors occurring around the same time, including regulatory validation, commercial expansion, funding, technological development, publication of ongoing clinical trials, and broader market interest.

‘Proactive Exploration’ is clearly exemplified by the Spasticity (ULS/LLS) line. As shown in the figure, this topic was already a top-ranked keyword in the earliest period (1983–1989), decades before its primary regulatory validation. This demonstrates that the technical knowledge base required to overcome high technological friction was established long before institutional formalization, with academic interest preceding market entry. As for ‘Reactive Catalysis’, it is perfectly represented by the NDO/OAB (Urinary) line. It first appears in the 2011–2013 period, concurrent with its initial FDA approval, and then demonstrates a sharp subsequent rise in rank. This pattern illustrates what we term “Reactive Catalysis”: research activity became more prominent around and after regulatory approval, although the temporal relationship does not establish that approval caused this change.

In the sensitivity analysis restricted to direct indication-related terms, 5 of the 9 evaluable indication groups (55.6%) showed prominent keywords before FDA approval, two were concurrent or slightly prior, and two appeared after approval. No direct Top-10 keyword was identified for platysma bands. Although the proportion of pre-approval signals was lower than in the broader mapping analysis (7/10), the pre-approval pattern remained evident for cervical dystonia, spasticity, pediatric cerebral palsy, chronic migraine, and hyperhidrosis. Looking across the entire period, we observe the BoNT innovation system maturing from foundational knowledge accumulation to anatomical and technical refinement. While each period is clustered independently, comparing the themes reveals a clear technology life-cycle consisting of three phases:

- Foundational phase (≈1983–2000): early research focused on building the core knowledge base, dominated by animal models and the initial clinical validation of Botox for early indications like blepharospasm [10]. The guiding questions centered on understanding the mechanism and potential application boundaries of the technology [11].
- Expansion phase (≈2000–2013): bracketed by major regulatory milestones for Botox in 2000 and 2002, this phase saw rapid diversification of the application landscape. Research themes expanded to include chronic pain, urology, and spasticity, directly mirroring the broadening regulatory profile and technological diffusion of Botox [12,13].
- Refinement phase (≈2013-present): the research frontier has transitioned from verifying efficacy to optimizing the technical workflow and reducing implementation friction. Research now emphasizes methodological precision and quality-focused themes, such as quantitative assessment and high-fidelity anatomical mapping tools (e.g., Sihler’s stain for neural distribution) [14,15].

Taken the evidence shows a temporal relationship within the innovation system: early, often off-label technical exploration precedes regulatory milestones, while shifts in research priorities are also observed after subsequent Botox approvals.

Recent trends (2020–2024) demonstrate a bifurcation in the innovation landscape. One major cluster centers on neurorehabilitative and functional restoration—covering spasticity, stroke, and cerebral palsy—while another emphasizes aesthetic and dermatological applications, including wrinkles, aesthetic surgery, and platysma-related rejuvenation. The simultaneous rise of ‘aesthetic’ and ‘neurofunctional’ terms indicates a growing convergence between cosmetic and restorative paradigms in BONT research, where aesthetic enhancement and neuromuscular modulation are increasingly viewed as complementary rather than separate domains.

Importantly, the chronological pattern observed across indications may help identify candidate areas of translational interest for future investigation. As most prior regulatory validations were preceded by a distinct period of concentrated academic attention, current emerging keywords, such as stroke, trigeminal neuralgia, xerostomia, and facial nerve paralysis, may therefore represent candidate translational areas warranting further clinical and regulatory investigation. These areas share common structural and technological barriers, suggesting high potential for near-term translational breakthroughs once these ambiguities are resolved.

From a broader perspective, these results show that technical knowledge accumulation, represented here by anatomical investigation, often precedes rather than simply follows regulatory milestones. This temporal pattern is consistent with a co-evolutionary relationship in which changes in the scientific knowledge base are observed both before and after regulatory milestones.

We proposed a possible explanation for the different lag patterns descriptively observed in Table 6: these patterns may be related to anatomical complexity (the Structural Complexity, C_sc_) and accessibility (the Non-therapeutic Market Pull, N_non-rx_).

First, comparatively long observed lags in Table 6 were seen for several indications involving structurally complex targets, which may partly reflect that this complexity creates high “technical noise” (technological uncertainty), necessitating decades for the technology to mature through deep, multi-layered systems. Indications such as Upper/Lower Limb Spasticity (~25-year lag) and Cervical Dystonia (~10-year lag) shown in Table 6 required decades to overcome the high implementation friction of deep, multi-layered musculoskeletal structures [16]. This extended timeline is also evident in Pediatric Spasticity, reflecting the amplified challenge of validating safety in smaller, developing physiologies, where the margin for error is minimal [17]. In this population, limited cooperation further increases implementation difficulty, demanding the transition from static anatomical targets to dynamic ones, and applying advanced guidance technologies (e.g., ultrasound) to ensure safety [18]. In these fields, the delay represents the necessary accumulation of knowledge required to evolve from theoretical identification to the image-guided precision required for standardized, safe clinical practice.

Second, comparatively shorter observed lags were seen for several superficial and sensory targets, with the relevant intervals in Table 6 falling approximately within the 2–6-year range, as their inherent accessibility removes structural ambiguity, thereby reducing the technical friction between discovery and regulatory approval. For indications like Chronic Migraine, Platysma Bands, and Hyperhidrosis, the anatomical targets present a lower C_sc_ compared to deep spasticity. Instead of requiring navigation through complex muscle layers, these targets are characterized by immediate surface accessibility (e.g., the platysma or intradermal sites for hyperhidrosis), or rely on palpation of sensory trigger points and fixed surface landmarks (e.g., migraine protocols) [19]. Unlike limb spasticity, where ‘blind’ localization has historically shown low accuracy without instrumental guidance (e.g., ultrasound/EMG), these superficial targets allowed clinical protocols to be codified and refined rapidly without a prolonged phase of anatomical visualization research [20,21].

Third, Table 6 shows minimal to near-zero observed separation for some visually standardized targets. Urinary indications (NDO/OAB) serve as a prime example of “concurrent translation”, where the integration of cross-disciplinary visual tools (e.g., cystoscopy) renders the internal muscle directly accessible, effectively neutralizing anatomical ambiguity and implementation friction [22]. Similarly, early indications like Blepharospasm and Strabismus benefited from the already established, detailed surgical knowledge within ophthalmology, allowing for rapid clinical adoption and institutional validation without a preceding proactive exploration phase [23].

Furthermore, observing the specific approved indications reveals a distinct pattern regarding the nature of the application and its corresponding market demand. Indications with a strict therapeutic requirement tended to show longer observed approval lags, as exemplified by the cluster ‘BONT for Dystonia and Dysphonia’ (Cluster #2, 1989–2000), and the corresponding approval for “Cervical dystonia”. Conversely, indications driven by a strong consumer market pull (i.e., a high aesthetic or non-therapeutic focus) tended to show shorter observed lags. This accelerated translation is represented by the approval of ‘Glabellar/Forehead/Lateral canthal lines (Cosmetic)’, and its closely aligned clusters ‘Aesthetic and Dermatological Applications of Botulinum Toxin A’ (Cluster #1, 2000–2002).

Therefore, we propose a conceptual hypothesis for anatomy-related technologies: regulatory validation lag may be longer for structurally complex targets and shorter for indications with stronger non-therapeutic market pull: the Regulatory Validation Lag (T_lag_) of such interventions may increase with their Structural Complexity (C_sc_) and may decrease with their Non-therapeutic Nature, acting as a proxy for market pull (N_non-rx_). That is, greater anatomical complexity may correspond to a longer observed lag, whereas a stronger non-therapeutic market orientation may correspond to a shorter observed lag. These hypothesized directional relationships can be summarized conceptually as:

$$T_{\mathrm{lag}}\;\propto \;\frac{C_{\mathrm{sc}}}{N_{\mathrm{non}-\mathrm{rx}}}$$

This notation is intended solely as illustrative conceptual shorthand rather than a quantitative model or predictive equation. The proportionality symbol is used only to summarize the hypothesized directional relationship and does not represent an empirically estimated or statistically tested proportional relationship. C_sc_ and N_non-rx_ were not operationalized according to predefined or independently verified criteria and were not statistically tested against T_lag_.

T_lag_ represents the Regulatory Validation Lag, referring here to the approximate temporal separation between the first prominent Top-10 appearance reported in Table 6 and the corresponding regulatory approval milestone. And C_sc_ represents the Structural Complexity of the target site (refers conceptually to depth and localization difficulty). High complexity may correspond to a longer translational lag. N_non-rx_ represents the degree of Non-therapeutic Nature of the indication. In this framework, N_non-rx_ can be interpreted as a proxy for “Market Demand Intensity”. Unlike therapeutic indications (e.g., spasticity or dystonia), which are affected by public health budgets and insurance reimbursement policies, non-therapeutic applications directly interface with the consumer market. This direct market focus generates a powerful “Market Pull” effect, providing stronger economic incentives for stakeholders to overcome regulatory hurdles and technical uncertainties. Consequently, while high structural complexity (C_sc_) acts as a technological friction barrier, high market demand (N_non-rx_) may correspond to an innovation accelerator, shortening the duration between initial research and final approval.

Regulatory timelines are also influenced by many factors besides anatomical complexity and market orientation, including trial feasibility, safety, disease prevalence, commercial strategy, reimbursement, competing products, sponsor investment, and regulatory pathways. C_sc_ and N_non-rx_ should therefore be viewed as two possible explanatory factors rather than a complete model of regulatory delay.

On a broader scale, our time-sliced scientometric framework offers a methodological approach for retrospectively identifying temporal relationships between scientific activity and regulatory milestones. For R&D planners, policymakers, and regulators, such analyses may help identify emerging scientific signals that warrant closer prospective monitoring. Instead of treating regulatory approvals as unpredictable exogenous shocks, stakeholders can monitor the structural evolution of knowledge networks over time. While this time-sliced approach could potentially be adapted to other fields, it is important to note that our specific conceptual framework is domain-specific. Using this anatomy-driven analysis as a proof-of-concept, we demonstrate how scientometrics, a macro-level framework, generates specific clinical insights. While it effectively characterizes anatomy-driven therapeutics (e.g., BoNT), it cannot be directly applied to systemic drugs. Any future attempt to model translational lag in systemic drugs, such as oral biologics, would require substituting structural complexity (C_sc_) with relevant systemic barriers like pharmacokinetic challenges or off-target toxicity. This suggests that studies in other therapeutic fields would need to define and test their own domain-specific barriers.

### 3.1. Future Directions: Exploratory Application

To illustrate the hypothesis-generating utility of the framework, we apply it to identify current BoNT research areas that may warrant future translational and regulatory attention. Rather than just identifying clinical hotspots, this framework may help identify areas that warrant future translational and regulatory attention.

In the most recent period (2021–2024; Table 3), keywords such as “facial nerve paralysis” and “facial palsy” exhibit a rise, which is accompanied by the emergence of a focused cluster on the treatment of facial motor disorders (Cluster #5, Table 5). Facial reanimation has shown a relatively slow translational trajectory. Its requirement for selective targeting of multiple facial muscles qualitatively corresponds to greater structural complexity C_sc_ within our conceptual shorthand. Recent trends suggest a technological convergence: injection strategies refined for aesthetic symmetry are being adapted for functional restoration, including Bell’s palsy and post-stroke facial asymmetry. Leveraging the established protocols from the commercialized aesthetic sector (representing an indirect N_non-rx_ spillover), the implementation friction for these functional disorders is reduced, suggesting continued translational development in this area.

The continued prominence of “trigeminal neuralgia” and “xerostomia” suggests that BoNT research is moving beyond the neuromuscular junction to include microscopic neural and glandular structures. These indications involve substantial anatomical and localization complexity, qualitatively corresponding to greater C_sc_ within the conceptual shorthand. Recent advances in image guidance may reduce some of these localization challenges. However, the maturation of ultrasound guidance, reflected by the “Anatomical Guides” cluster (Cluster #9, Table 5), acts as a crucial uncertainty-reducing mechanism. As these imaging technologies reduce localization difficulties, neuropathic pain indications may represent an area of continued translational development, mimicking the trajectory observed in chronic migraine.

Although spasticity is an established indication, the re-emergence of “stroke” (ranked #7 in 2021–2024) together with signals from the “Real-World Evidence” cluster (Cluster #7) suggests a potential shift in the innovation system: moving from efficacy validation to the refinement of the workflow. Earlier work focused mainly on whether BoNT improves spasticity, and current trends put emphasis on how to optimize outcomes for specific post-stroke phenotypes through more evidence. In parallel, the appearance of DaxibotulinumtoxinA in recent clusters points to a growing interest in long-acting formulations. For R&D managers, this signals that the next phase of spasticity innovation will be driven not only by discovering new anatomical targets, but also by compounding novel drug formulations with image-guided precision to further eliminate implementation friction.

Beyond thematic trends, our framework highlights several candidate indications showing sustained scientific and translational interest.

Benign masseter hypertrophy emerges as one candidate indication showing a mature and sustained research base. The literature shows a mature research base, including clusters centered on masseter anatomy and treatment (Cluster #6, 2019–2020) and broader work on masticatory muscles (Cluster #7, 2020–2021). Recent studies published after our observation period provide additional context for the continued development of this field. A 2025 review summarized the growing evidence for BoNT/A in masseter muscle hypertrophy, including its effects on muscle volume, injection techniques, and clinical outcomes, while also noting the lack of consensus on dosing [24]. More recently, a 2026 pilot study illustrated the increasing use of quantitative morphometric assessment in this area. These two studies were not included in our bibliometric dataset and are cited as evidence of continued scientific development [25]. The masseter is large, superficial, and relatively easy to localize, characteristics that qualitatively correspond to lower structural complexity within the conceptual shorthand. At the same time, the strong consumer-facing aesthetic orientation of lower-face contouring qualitatively corresponds to stronger non-therapeutic market pull.

Facial synkinesis related to facial nerve paralysis represents another candidate translational area identified by the recent scientometric signal. “Facial nerve paralysis” has been a high-frequency keyword over the past decade, and the most recent period (2021–2024) includes a dedicated cluster focused on facial palsy-related conditions (Cluster #5). Compared with the masseter, facial targets require better selectivity and carry a higher risk of off-target functional changes, qualitatively corresponding to greater structural complexity within the conceptual shorthand. However, the growing use of established aesthetic symmetry techniques to support functional recovery may provide transferable procedural experience for this indication. Existing clinical recommendations also highlights the importance of detailed functional anatomy and individualized muscle selection in facial palsy, supporting the technical complexity of this indication [26].

Trigeminal neuralgia represents a further candidate area characterized by sustained scientific interest but greater anatomical and clinical complexity. The keyword spiked as a notable focus earlier in the timeline, and pain-related clusters have persisted across multiple periods, indicating sustained interest. The main implementation challenge involves deep and anatomically sensitive targets with limited reproducibility, characteristics that qualitatively correspond to greater structural complexity. Recent growth in image guidance and anatomical mapping may improve localization and procedural reproducibility, supporting continued translational investigation in this area [27].

Importantly, scientometric prominence could not be interpreted as the only evidence of clinical readiness. A growing literature may reflect scientific interest or technical development without establishing efficacy, safety, optimal dosing, or patient selection. This distinction is relevant in areas such as orofacial and neuropathic pain, where expanding BoNT use coexists with substantial heterogeneity in clinical protocols and evidence quality [28]. Therefore, the candidate indications identified here should be viewed as areas for further investigation rather than as evidence supporting clinical adoption.

While these specific projections focus on BoNT, this proof-of-concept demonstrates the same time-sliced approach could be adapted to other biopharmaceutical fields. By substituting anatomical complexity (C_sc_) with relevant domain-specific barriers (e.g., delivery vehicle instability in mRNA therapeutics, or off-target toxicity in antibody-drug conjugates), future studies could test whether similar variables can be combined with time-resolved scientometric signals to study temporal relationships in other biopharmaceutical fields.

### 3.2. Limitations

First, we centered on applied clinical anatomy. We grouped injection techniques, guidance methods (e.g., ultrasound, EMG), and surface landmarks under this heading for a practical reason: unlike surgery, where tissues are exposed, BoNT injections rely on accessing internal targets through intact skin [29]. As a result, in this field, a muscle is defined not only by its origin and insertion, but also by its depth relative to the skin, the preferred injection trajectory, and its sonographic appearance [30]. To describe morphology without these parameters would fail to capture the translational nature of the field. Therefore, we treat delivery techniques not as separate methodological steps, but as intrinsic components of ‘applied clinical anatomy’. In this context, including ultrasound and EMG data is not a concession; it is a necessity for accurately depicting how anatomical knowledge is applied in practice.

A further limitation concerns the breadth of the search strategy. Our conceptual definition of applied clinical anatomy includes guidance techniques, injection depth, and related procedural factors, whereas the database search was anchored to explicit anatomical terms such as anatomy, landmarks, and target muscles. Therefore, studies focused mainly on ultrasound, EMG, or other guidance methods may have been missed if they did not also contain one of these anatomical terms. The present corpus should therefore be interpreted as anatomy-anchored BoNT literature rather than an exhaustive collection of all procedural or guidance-related BoNT studies.

Second, we used the U.S. FDA approval timeline for Botox as our temporal anchor. We acknowledge this creates a U.S.- and product-centric frame of reference. However, these approvals are used only as a consistent temporal anchor and should not be interpreted as representing the complete regulatory history of the BoNT class. Crucially, our clustering results (Table 5) confirm that this choice did not obscure data from other brands. Distinct clusters emerged for non-Botox formulations, including Myobloc, Dysport, and Daxxify, independent of the Botox signal. This demonstrates that while our timeline is based on one flagship product, our network analysis retains high enough resolution to distinguish specific formulation dynamics from broader class-wide trends. The regulatory eras also differed in duration because they were defined by FDA approval milestones rather than fixed calendar intervals. Longer periods may provide greater opportunity for keywords or clusters to emerge. Therefore, comparisons across eras should be interpreted as differences in thematic prominence and temporal alignment rather than as direct comparisons of publication volume or cluster accumulation.

Importantly, the present analysis is retrospective and was structured around known regulatory milestones. It therefore identifies temporal associations and candidate scientific signals but does not establish prospective predictive performance. Validation using future regulatory events will be required before the framework can be considered a forecasting tool.

Third, relying exclusively on Web of Science and Scopus inherently introduces publication and indexing biases. Proprietary industry R&D, negative or discontinued trials, and informal scientific discourse are largely absent from these databases. Consequently, our scientometric signals capture only the visible, peer-reviewed domain, which would inevitably skew toward active and successful trajectories over abandoned efforts. The lack of an observable signal in our dataset should therefore not be equated with an absence of underlying R&D activity.

## 4. Conclusions

This scientometric analysis reveals a co-evolutionary relationship between technical knowledge accumulation and the regulatory milestones for Botox^®^. In most indications, scientific attention functioned as proactive exploration, establishing technical feasibility years or even decades before formal approval. In some areas, research activity became more prominent around or after regulatory milestones, particularly in studies focused on technical refinement and safety optimization. Furthermore, the duration of the translational lag between research peaks and approvals may be related to the interplay between technological complexity (C_sc_) and market demand intensity (N_non-rx_).

Time-resolved scientometrics maps the temporal alignment between literature signals and regulatory milestones in BoNT. While research activity frequently preceded formal approvals, other domains intensified only post-approval. These retrospective patterns provide a hypothesis-generating framework for characterizing translational trajectories. However, establishing genuine regulatory forecasting utility will require further prospective validation.

## 5. Materials and Methods

### 5.1. Data Sources and Screening Process

We conducted a literature search on 1 October 2025, using the Web of Science and Scopus databases (search queries detailed in Table 7). 2025 publications were excluded because the search was conducted before the end of 2025. We intentionally designed a broad search strategy that included generic terms, brand names (e.g., Botox), and specific formulations, rather than restricting the scope to a single product.

This inclusive approach was driven by two key factors. First, to ensure scientometric completeness and avoid recall bias, as early literature often uses specific and generic names interchangeably [31]. Second, although different BoNT serotypes and formulations are pharmacologically distinct and not interchangeable in terms of dosing, they share a common mechanism of action at the neuromuscular junction and are applied to the same target muscles and glands; thus, the underlying injection anatomy is largely serotype-agnostic at the level of gross target localization [32].

Crucially, we employed a broad definition of ‘anatomy-related’ literature. We view the field as evolving from descriptive anatomy (classical studies of static structure) to applied clinical anatomy: the application of this knowledge in a clinical context, covering injection localization, guidance techniques, and safety margins [33].

Within the retrieved corpus, anatomy-related research could include the following content when the studies also met the anatomical search criteria: (1) direct anatomical studies (e.g., cadavers, nerve staining, anatomical landmarks); (2) studies or comparisons of guidance techniques (e.g., ultrasound, EMG); (3) studies or comparisons of specific injection sites, depths, or patterns; (4) assessments of anatomical morphological changes via imaging (e.g., MRI or CT evaluation of muscle volume); (5) explorations of relationships between anatomical structures and specific efficacy or complications; and (6) reviews, consensus statements, or guidelines centered on these anatomy-related applications.

Our research question was not limited to traditional, cadaver-based anatomy; rather, it tried to identify how anatomical knowledge and anatomy-driven techniques have influenced clinical practice, patient safety, medical research, and effectiveness trends in BoNT injection. Accordingly, we intentionally adopted a broad, a priori definition of ‘anatomy-related’ literature that includes both classical anatomical studies and clinical or methodological studies whose intervention or primary comparison is anatomical. This conceptual expansion is deliberate, since it captures the complete pathway from anatomical discovery to applied clinic. Therefore, this method aligns with our objective of mapping anatomy-driven hotspots that have shaped practice milestones.

Using the query, the initial retrieval resulted in 2233 records from WoS and 4945 from Scopus. To ensure that the process was systematic and reproducible, the PRISMA guidelines were followed. These two databases were merged, and 1643 papers were de-duplicated using DOI/Title double matching. After the identification phase, papers were filtered to exclude non-English publications and non-article or non-review types. These steps ensured the quality and consistency of the data for further analysis. As a result, 4648 papers were retained.

In Stage 2, two reviewers independently examined the titles and abstracts of the 4648 records to determine their close relevance to Botox-related research. This screening excluded 1591 irrelevant articles. The level of agreement between the reviewers was high (Cohen’s Kappa of 0.744, 95% CI = 0.70–0.77). Any disagreements on inclusion were resolved through discussion with a third, senior author. This yielded 3057 papers for the next stage.

**Figure 2:**
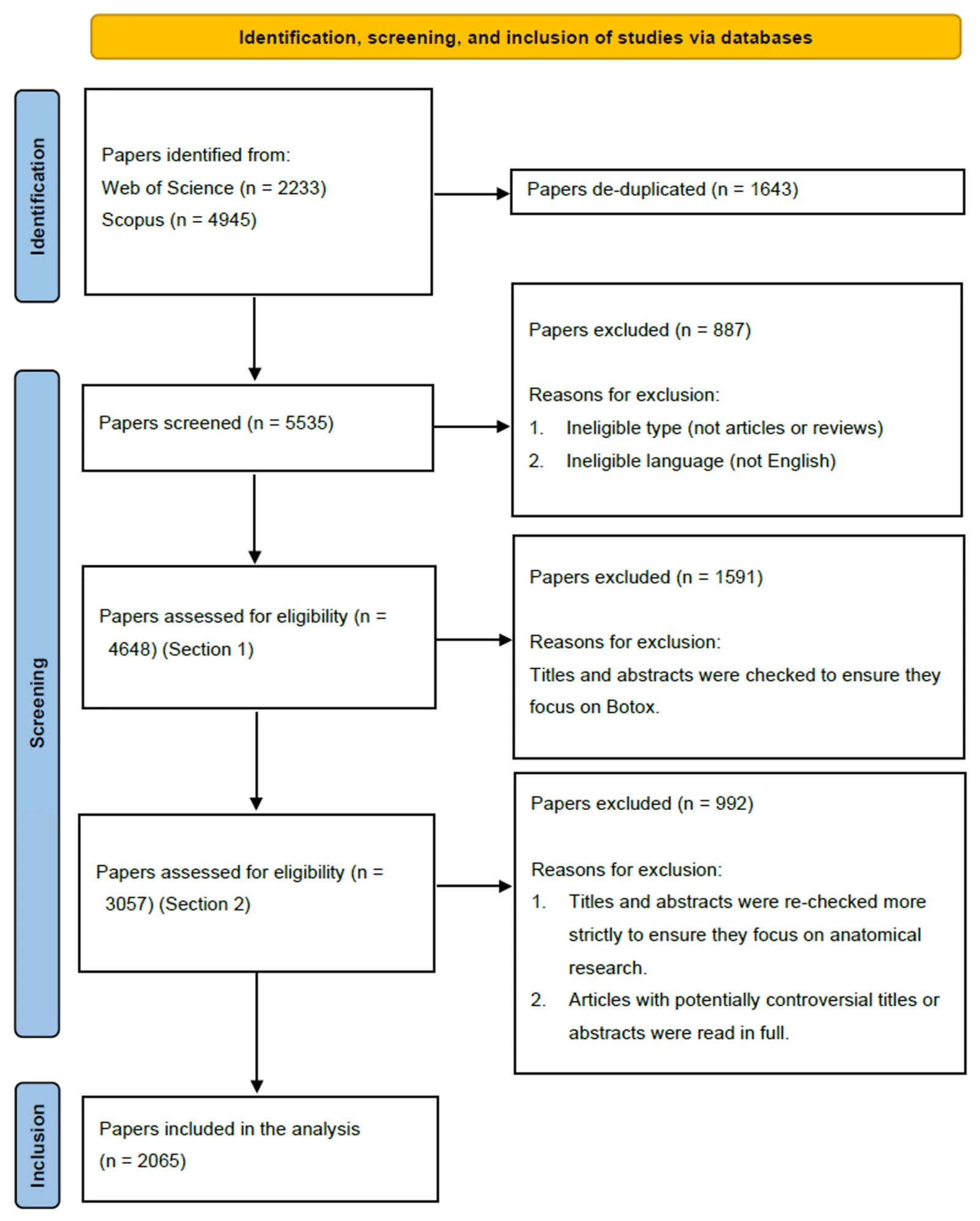
Identification, screening, and inclusion process.

In Stage 3, the titles and abstracts of the remaining papers were re-examined to ensure they were closely related to anatomical research. As indicated in Figure 2, articles with potentially controversial titles or abstracts were read in full. This final screening, jointly discussed by all authors to reach a consensus, excluded 992 articles. As a result, a final corpus of 2065 articles was included for subsequent analysis.

The reason why we included two sections of examination was to maximize the ‘signal-to-noise’ ratio: that is, the proportion of ‘relevant to irrelevant’ studies.

### 5.2. Research Tools

To develop a detailed intellectual map of the field, data such as titles, abstracts, keywords, and related metadata were collected from the WoS and Scopus databases. This data was then stored in both a CSV file created with Microsoft Excel 2019 (Microsoft, Redmond, WA, USA) and a standard TXT file.

Our analytical strategy adopted a dynamic analysis using CiteSpace (version 6.4.1.0, Advanced; Chaomei Chen, Drexel University, Philadelphia, PA, USA) to view emergent research hotspots [34].

### 5.3. Research Process

CiteSpace (with a slice length of one year) was employed to perform the keyword analysis. In analyzing the literature on anatomy-related topics in Botox research, the g-index selection criterion in CiteSpace was applied, as it is widely used in scientometric research and allows for cross-study comparability. This method also helps to reduce the number of nodes, thereby decreasing network density, enhancing visual clarity, and maintaining representativeness [35].

To determine the optimal value for k, sensitivity analysis was performed by testing various thresholds on every FDA approval period represented in Table 1. In regulatory terms, FDA approvals are granted to specific BoNT formulations rather than to the toxin family as a whole. For this study, we extracted the approval dates of Botox^®^/Botox^®^ Cosmetic for each therapeutic and aesthetic indication from official FDA labelling and available regulatory documents. These dates were used as external marks to allocate the BoNT literature into different regulatory eras. Importantly, our scientometric corpus includes anatomy-related BoNT studies, whereas the FDA timeline is based specifically on Botox and is therefore used as a consistent temporal anchor rather than as a surrogate for the regulatory history of the entire BoNT class.

After setting the node type to ‘keyword’, we found that the relative size of the largest connected component (LCC) peaked when k was set to 25 in most periods (15 out of all 16 periods; Strabismus and Blepharospasm were merged into one period). Consequently, the g-index with k = 25 was selected as the final parameter to ensure that our analysis was based on the most coherent and representative core knowledge network, while avoiding irrelevant connections [4].

Other parameters of CiteSpace were set as follows: (1) the time slicing was configured based on the FDA approval period, with one year per slice; (2) for term sources, we selected titles, abstracts, author keywords, and Keywords Plus; (3) the Link Strength was set to Cosine, and the Link Scope was set to Within Slices; (4) no pruning methods were applied; and (5) finally, the visualizations were generated using the ‘cluster view-static’ and ‘show merged network’ options. 

Microsoft Excel (version 2019, Microsoft Corp., Redmond, WA, USA) and IBM SPSS Statistics (Version 22.0; IBM Corp., Armonk, NY, USA) handled charting and descriptive statistics.

## Figures and Tables

**Table 1:** The FDA approval timeline.

FDA App. Date	Indication/Use	Population	Brand/Division
29 December 1989	Strabismus	Adults	Oculinum
30 December 1989	Blepharospasm	Adults	Oculinum
21 December 2000	Cervical dystonia	Adults	Botox
4 December 2002	Glabellar lines (GL)	Adults	Botox Cosmetic
19 July 2004	Primary axillary hyperhidrosis (PAH)	Adults	Botox
9 March 2010	Upper limb spasticity (ULS)	Adults	Botox
15 October 2010	Chronic migraine prevention (CM Prev)	Adults	Botox
24 August 2011	Urinary incontinence due to neurogenic detrusor overactivity (NDO)	Adults	Botox
18 January 2013	Overactive bladder (OAB)	Adults	Botox
11 September 2013	Lateral canthal lines (LCL)	Adults	Botox Cosmetic
22 January 2016	Lower limb spasticity (LLS)	Adults	Botox
3 October 2017	Forehead lines (FL)	Adults	Botox Cosmetic
21 June 2019	Upper limb spasticity (Peds ULS)	Pediatric (2–17 yr)	Botox
24 October 2019	Lower limb spasticity (excluding cerebral palsy) (Peds LLS)	Pediatric (2–17 yr)	Botox
9 July 2020	Pediatric spasticity including cerebral palsy (Peds Spasticity incl. CP)	Pediatric (2–17 yr)	Botox
10 February 2021	Neurogenic detrusor overactivity (Peds NDO)	Pediatric (≥5 yr)	Botox
18 October 2024	Platysma bands (PB)	Adults	Botox Cosmetic

**Table 2:** The top 10 keywords of different FDA approval periods.

**FDA Appr.** **Keyword Ranking**	**1983.01–1989.12**	**1989.12–2000.12**	**2000.12–2002.12**	**2002.12–2004.07**	**2004.07–2010.03**	**2010.03–2010.10**	**2010.10–2011.08**	**2011.08–2013.01**
#1	botulinum toxin	botulinum toxin	human	botulinum toxin type A	botulinum toxin type A	botulinum toxin	botulinum toxin	botulinum toxin
#2	human	human	botulinum toxin type A	human	human	botulinum toxin	botulinum toxin	botulinum toxin type A
#3	facial muscles	article	article	article	botulinum toxin	human	human	human
#4	spasm	botulinum toxin type A	botulinum toxin	priority journal	article	article	article	article
#5	blepharospasm	female	female	injection site	priority journal	female	adult	priority journal
#6	Male	male	adult	botulinum toxin	female	adult	female	drug efficacy
#7	female	adult	priority journal	female	neuromuscular agents	male	priority journal	female
#8	aged	facial muscle	male	treatment outcome	adult	drug efficacy	drug efficacy	adult
#9	eyelid diseases	priority journal	injection site	adult	clinical trial	treatment outcome	male	injection site pain
#10	adult	blepharospasm	clinical trial	clinical trial	drug efficacy	priority journal	injection site	male
**FDA Appr.** **Keyword Ranking**	**2013.01–2013.09**	**2013.09–2016.01**	**2016.01–2017.10**	**2017.10–2019.06**	**2019.06–2019.10**	**2019.10–2020.07**	**2020.07–2021.02**	**2021.02–2024.10**
#1	botulinum toxin	botulinum toxin type A	botulinum toxin	botulinum toxin	botulinum toxin	botulinum toxin	botulinum toxin	botulinum toxin
#2	botulinum toxin type A	botulinum toxin	botulinum toxin type A	botulinum toxin type A	botulinum toxin type A	botulinum toxin type A	botulinum toxin type A	botulinum toxin type A
#3	human	human	human	human	human	human	human	human
#4	priority journal	article	injections	article	article	article	female	article
#5	female	female	article	injections	female	female	article	adult
#6	article	injections	female	female	male	adult	adult	female
#7	drug efficacy	male	male	adult	adult	male	male	male
#8	double blind	adult	adult	male	priority journal	priority journal	treatment outcome	double blind
#9	treatment outcome	priority journal	neuromuscular agents	priority journal	injections	double blind	double blind	controlled study
#10	injection site pain	treatment outcome	priority journal	drug efficacy	middle aged	treatment outcome	muscle relaxant agent	drug efficacy

**Table 3:** The top 10 cleansed keywords.

**FDA Appr.** **Keyword Ranking**	**1983.01–1989.12**	**1989.12–2000.12**	**2000.12–2002.12**	**2002.12–2004.07**	**2004.07–2010.03**	**2010.03–2010.10**	**2010.10–2011.08**	**2011.08–2013.01**
#1	spasticity	blepharospasm	spasticity	ptosis	ptosis	ptosis	spasticity	ptosis
#2	blepharospasm	spasticity	ptosis	skin aging	dysphagia	cervical dystonia	cervical dystonia	cervical dystonia
#3	eyelid diseases	hemifacial spasm	dystonia	spasticity	spasticity	blepharospasm	ptosis	dysphagia
#4	hemifacial spasm	ptosis	skin aging	dysphagia	blepharospasm	spasticity	dysphagia	blepharospasm
#5	paralysis	dystonia	dysphagia	dystonia	skin aging	dysphagia	blepharospasm	spasticity
#6	facial paralysis	dysphonia	anus fissure	esthetics	cervical dystonia	skin aging	cosmetic techniques	cerebral palsy
#7	dystonia	torticollis	hyperhidrosis	cerebral palsy	hyperhidrosis	hyperhidrosis	hyperhidrosis	overactive bladder
#8	meige syndrome	dysphagia	cervical dystonia	hyperhidrosis	dystonia	esthetic surgery	skin aging	urinary incontinence
#9	blepharoptosis	esthetic surgery	cerebral palsy	migraine	wrinkles	glabellar lines	xerostomia	xerostomia
#10	movement disorders	skin aging	strabismus	rejuvenation	hemifacial spasm	hypertrophy	overactive bladder	hyperhidrosis
**FDA Appr.** **Keyword Ranking**	**2013.01–2013.09**	**2013.09–2016.01**	**2016.01–2017.10**	**2017.10–2019.06**	**2019.06–2019.10**	**2019.10–2020.07**	**2020.07–2021.02**	**2021.02–2024.10**
#1	ptosis	ptosis	spasticity	spasticity	spasticity	ptosis	spasticity	spasticity
#2	dysphagia	dysphagia	dysphagia	ptosis	ptosis	spasticity	cervical dystonia	cervical dystonia
#3	overactive bladder	cervical dystonia	cervical dystonia	cerebral palsy	migraine	cervical dystonia	dysphagia	dysphagia
#4	cervical dystonia	xerostomia	ptosis	cervical dystonia	cerebral palsy	esthetic surgery	ptosis	ptosis
#5	urinary incontinence	cerebral palsy	cerebral palsy	esthetic surgery	skin aging	cosmetic techniques	esthetic surgery	cerebral palsy
#6	spasticity	spasticity	hyperhidrosis	cosmetic techniques	cervical dystonia	skin aging	cerebral palsy	migraine
#7	xerostomia	skin aging	esthetic surgery	skin aging	migraine disorders	migraine	skin aging	stroke
#8	facial nerve paralysis	facial nerve paralysis	trigeminal neuralgia	transformed migraine	chronic migraine	dysphagia	cosmetic techniques	wrinkles
#9	urinary retention	wrinkles	stroke	stroke	facial nerve paralysis	transformed migraine	migraine	hemifacial spasm
#10	cerebral palsy	hyperhidrosis	glabellar lines	migraine disorders	dystonia	chronic migraine	wrinkles	facial nerve paralysis

**Table 4:** Keyword grouping map and rationale.

ID	FDA Approval Content	The Cleansed Keywords	Reason
1	Glabellar lines (GL); Lateral canthal lines (LCL); Forehead lines (FL); Platysma bands (PB)	wrinkles; glabellar lines; ptosis (side effect); blepharoptosis (side effect)	The keywords are highly and specifically related to the approval content. The side effects are closely related to the approval content.
2	Upper limb spasticity (ULS); Lower limb spasticity (LLS); Upper limb spasticity (Peds ULS); Lower limb spasticity excluding cerebral palsy (Peds LLS)	spasticity	The keyword is highly and specifically related to the approval content.
3	Pediatric spasticity including cerebral palsy (Peds Spasticity incl. CP)	cerebral palsy	The keyword is the same as the approval content.
4	Urinary incontinence due to neurogenic detrusor overactivity (NDO); Overactive bladder (OAB); Neurogenic detrusor overactivity (Peds NDO)	overactive bladder; urinary incontinence; urinary retention (side effect)	The keywords are highly and specifically related to the approval content. The side effect is closely related to the approval content.
5	Cervical dystonia	cervical dystonia; dystonia; torticollis; dysphagia (side effect); dysphonia (side effect)	The keywords are highly and specifically related to the approval content. The side effects are closely related to the approval content.
6	Blepharospasm	blepharospasm; meige syndrome (highly related syndrome); hemifacial spasm	The keyword is highly and specifically related to the approval content. The syndrome is closely related to the approval content.
7	Chronic migraine prevention (CM Prev)	chronic migraine; migraine; migraine disorders; transformed migraine	The keywords are highly and specifically related to the approval content.
8	Strabismus	strabismus	The keyword is the same as the approval content.
9	Primary axillary hyperhidrosis (PAH)	hyperhidrosis	The keyword is the same as the approval content.
10	Excluded Keywords	Group A: anus fissure, hypertrophy, trigeminal neuralgia, xerostomia. Group B: movement disorders, eyelid diseases, cosmetic techniques, esthetic surgery, esthetics, facial nerve paralysis, facial paralysis, paralysis, rejuvenation, skin aging, stroke.	The keywords in Group A are not closely related to the Botox FDA approval content. The keywords in Group B are overly general or refer to several different FDA approval groups.

**Table 5:** The keyword clusters of different FDA approval periods.

**Cluster Names/** **FDA Approval Date**	**1983.01–1989.12**	**1989.12–2000.12**	**2000.12–2002.12**	**2002.12–2004.07**	**2004.07–2010.03**	**2010.03–2010.10**	**2010.10–2011.08**	**2011.08–2013.01**
Cluster #0	Clinical Studies for Blepharospasm	Possible Applications of Botulinum Toxin Type A	Chronic Pain Management	Clinical Investigation of Botulinum Toxin Type B	Clinical Investigation of Botulinum Toxin Type B and Tolerability	Possible Applications of Botulinum Toxin Type A	Possible Applications of Botulinum Toxin Type A	Side Effects of BONT
Cluster #1	Preclinical Animal Models Research on BONT	Clinical Studies for Blepharospasm	Aesthetic and Dermatological Applications of Botulinum Toxin A	Chronic Migraine Treatment	Aesthetic and Dermatological Applications of BONT	Aesthetic and Dermatological Applications of BONT	Neurological Applications of BONT and Side Effects	Methodology for Pain and Outcome Assessment
Cluster #2	Biochemical and Structural Characterization of BONT	BONT for Dystonia and Dysphonia	Aesthetic and Dermatological Applications of Botulinum Toxin B	Possible Applications of Botulinum Toxin Type A	Side Effects of BONT	Clinical Investigation of Botulinum Toxin Type B and Tolerability	Clinical Investigation of Botulinum Toxin Type B	Possible Applications of Botulinum Toxin Type A
Cluster #3	Treatment of Facial Nerve Paralysis Complications	Biochemical and Structural Characterization of BONT	BONT for Dystonia and Dysphonia	Aesthetic and Dermatological Applications of Botulinum Toxin A	Possible Applications of Botulinum Toxin Type A	BONT for Spasticity	Pain Assessment within the BONT Contexts	Urological Use of Botulinum Toxin
Cluster #4	BONT for Dystonia and Dysphonia	Case Reports on Emerging Uses of BONT	Comparative Studies of Injection Techniques	Side Effects and Concomitant Analgesic Use	Urological Use of Botulinum Toxin	Dose–Response Studies on BONT for Pain and Tolerability	Aesthetic and Dermatological Applications of BONT	Aesthetic and Dermatological Applications of BONT
Cluster #5	Possible Applications of Botulinum Toxin Type A	Studies of BONT for Anal Fissure	Studies of BONT for Anal Fissure	Aesthetic and Dermatological Applications of Botulinum Toxin B	Neurological Applications of BONT	Techniques for Reducing Injection Pain in Aesthetic Procedures	Rating Scales Use within the BONT Contexts	Efficacy and Patient Satisfaction of BONT
Cluster #6	Bio-Investigation of Botulinum Toxin Type B	Methodology Use of Botulinum Toxin	Placebo-Controlled Studies on Botulinum Toxin for Spasticity	Randomized Controlled Trials for Chronic Pain	BONT Impact on the Quality of Life	Side Effects of BONT and Impact on Quality of Life	Reducing Injection Pain during Aesthetic Treatments	Reviews of BONT Effects
Cluster #7	Methodology Use of Botulinum Toxin	Preclinical Animal Models Research on BONT	Molecular Structure and Mechanism of BONT	Urological Use of Botulinum Toxin	BONT Treatment in Children	Using Pain Assessment for Analyzing the Clinical Effectiveness and Duration of BONT	Long-term Follow-up Studies	
Cluster #8	Case Reports on Emerging Uses of BONT	Aesthetic and Dermatological Applications of Botulinum Toxin	BONT Use under Psychiatric Contexts	Efficacy and Safety of Botulinum Toxin Type B	OnabotulinumtoxinA Applications and Side Effects		Aesthetic Injection Comfort	
Cluster #9	Surgical vs. Pharmacological Treatments for Benign Essential Blepharospasm		Urological Use of Botulinum Toxin	Methodological Studies of BONT	Reducing Injection Pain during Aesthetic Treatments		Urological Use of Botulinum Toxin	
Cluster #10							Assessing BONT Analgesic Efficacy	
**Cluster Names/FDA Approval Date**	**2013.01–2013.09**	**2013.09–2016.01**	**2016.01–2017.10**	**2017.10–2019.06**	**2019.06–2019.10**	**2019.10–2020.07**	**2020.07–2021.02**	**2021.02–2024.10**
Cluster #0	Urological Use of Botulinum Toxin	Side Effects from Placebo-Controlled Trials	Possible Applications of Botulinum Toxin Type A	Aesthetic Applications of Botulinum Toxin for Upper Facial Lines	Chronic Migraine and Aesthetic Research of OnabotulinumtoxinA	Randomized Controlled Trials in Aesthetics	Clinical Trials for Spasticity and Cervical Dystonia	Neurological Applications of BONT
Cluster #1	Long-Term Efficacy and Safety and Associated Side Effects	Diverse Applications of Onabotulinumtoxin A and Impact on Quality of Life	Treatment of Spasticity	Mechanism of Action and its Application in Spasticity	Chronic Migraine Treatment and Associated Side Effects	Chronic Migraine Clinical Trials Focusing on Side Effects and Quality of Life	Research about Abobotulinumtoxina	Aesthetic Applications for Full-Face, Lower-Face, and Neck
Cluster #2	Aesthetic and Neurological Applications of BONT	Aesthetic Trials for Glabellar and Crow’s Feet Lines	Reviews on Efficacy, Safety, and Side Effects	Chronic Migraine Treatment	Objective and Quantitative Assessment in Facial Aesthetics	Neurological Applications of BONT	Reviews of BONT Effects	Systematic Reviews and Meta-Analyses
Cluster #3	Efficacy and Patient Satisfaction of BONT	Possible Applications of Botulinum Toxin Type A	Clinical Studies for Chronic Pain and Impact on Quality of Life	Side Effects and Effect on Quality of Life	Clinical Trials for Spasticity in Pediatrics and Adolescents	Pediatric Applications for Spasticity and Cerebral Palsy	Chronic Migraine Treatment	Research on New Formulations (DaxibotulinumtoxinA, RelabotulinumtoxinA) for Aesthetics and Spasticity
Cluster #4	Treatment of Facial Nerve Paralysis Complications	Treatment and Pathophysiology of Facial Nerve Paralysis Complications	Aesthetic Applications of AbobotulinumtoxinA (Dysport)	Methodology for Assessing Clinical Outcomes	Chronic Pain Management	Systematic Reviews on BONT	Aesthetic Applications for Full-Face, Lower-Face, and Neck	Precise Anatomical Research (Sihler’s Stain, Neural Distribution)
Cluster #5	Systematic Reviews of BONT	Treatment of Spasticity and Associated Pain	Management of Post-Stroke Upper Limb Spasticity	Management of Post-Stroke Upper Limb Spasticity	Patient Satisfaction of Aesthetic Treatment	Neurological Applications of BONT	Treatment of Facial Motor Disorders	Treatment of Facial Palsy and Hemifacial Spasm
Cluster #6	BONT for Spasticity	Chronic Migraine Treatment	Analgesic Use of BONT for Orofacial Pain	Systematic Reviews on BONT	Assessment of Adverse Events	Anatomy, Histology, and Treatment of the Masseter Muscle	Outcome Assessment Scoring Systems, Severity and Placebo Control	Merging Surgical and Procedural Applications
Cluster #7		Pediatric Trials of BONT	Animal Models Research BONT	Dermatology Applications and Patient Satisfaction		Management of Chronic Pain and Associated Depression	Studies of Masticatory Muscles	Real-World Evidence
Cluster #8		New Formulations/Delivery Methods	Side Effects of BONT	Neurological Applications of BONT		Comparative Studies of Injection Techniques	Safety of Spasticity Treatment	Aesthetic Treatment of Upper Facial Lines
Cluster #9							DaxibotulinumtoxinA for Aesthetic Treatment	Anatomical Guides and Injection Techniques for Lower Limb Spasticity
Cluster #10								Safety of Spasticity Treatment in Clinical Practice
Cluster #11								DaxibotulinumtoxinA for Aesthetic Treatment

**Table 6:** Comparison between FDA approval dates and the first Top-10 appearance of corresponding indication-related keywords.

Indication Group	Representative Keywords	First Prominent Appearance (Top-10) Period	FDA Approval Date	Temporal Relationship
Strabismus	strabismus	2000.12–2002.12	1989.12	Appeared after approval
Blepharospasm	blepharospasm	1983.01–1989.12	1989.12	Concurrent to slightly prior
Cervical dystonia	dystonia, cervical dystonia, torticollis	1989.12–2000.12	2000.12	Preceded approval by ~10 years
Glabellar/Forehead/Lateral canthal lines (Cosmetic)	ptosis, skin aging, wrinkles	2000.12–2002.12	2002.12	Immediate pre-approval appearance
Primary axillary hyperhidrosis (PAH)	hyperhidrosis	2002.12–2004.07	2004.07	Appeared shortly before approval
Upper/Lower limb spasticity (ULS/LLS)	spasticity	1983.01–1989.12	2010.03/2016.01	Clearly preceded approval (≈20–25 years earlier)
Pediatric spasticity (including CP)	cerebral palsy	2010.10–2011.08	2019.06	Preceded approval by ~8 years
Chronic migraine	migraine, chronic migraine	2002.12–2004.07	2010.10	Preceded approval by ~6 years
Neurogenic/Overactive bladder (NDO/OAB)	overactive bladder, urinary incontinence	2011.08–2013.01	2011.08	Concurrent with approval (timing aligned)
Platysma bands (Cosmetic)	esthetic surgery, wrinkles	2019.10–2020.07	2024.10	~4–5 years before approval

**Table 7:** The query and the explanations.

	Query	Explanations
Web of Science	TS = (“botulinum toxin*” OR botox OR onabotulinumtoxinA OR abobotulinumtoxinA OR incobotulinumtoxinA OR rimabotulinumtoxinB OR daxibotulinumtoxinA OR “botulinum neurotoxin*”) AND TS = (anatom* OR dissection OR cadaver* OR “facial muscle*” OR “muscle target*” OR “injection point*” OR “injection site*” OR topography OR morphology OR landmark*)	1. TS = /TITLE-ABS-KEY was used to ensure the keywords occurred in both Titles, Abstracts, and Keywords (Keyword Plus included). 2. The query included two components: the first line contained keywords related to BoNT and their major formulations (including generic terms and trade names such as Botox), and the second line contained keywords related to anatomical and injection-related research.
Scopus	TITLE-ABS-KEY (“botulinum toxin*” OR botox OR onabotulinumtoxinA OR abobotulinumtoxinA OR incobotulinumtoxinA OR rimabotulinumtoxinB OR daxibotulinumtoxinA OR “botulinum neurotoxin*”) AND TITLE-ABS-KEY (anatom* OR dissection OR cadaver* OR “facial muscle*” OR “muscle target*” OR “injection point*” OR “injection site*” OR topography OR morphology OR landmark*)	

## Data Availability

The bibliometric data used in this study were obtained from the Web of Science Core Collection and publicly available regulatory information from the U.S. Food and Drug Administration (FDA). The processed data supporting the findings of this study are available from the corresponding author upon reasonable request.

## Abbreviations

The following abbreviations are used in this manuscript:

BoNT	Botulinum neurotoxin
FDA	Food and Drug Administration
PRISMA	Preferred Reporting Items for Systematic Reviews and Meta-Analyses
WoS	Web of Science
LCC	Largest connected component
IRR	Inter-rater reliability
EMG	Electromyography
GL	Glabellar lines
LCL	Lateral canthal lines
FL	Forehead lines
PB	Platysma bands
ULS	Upper limb spasticity
LLS	Lower limb spasticity
PAH	Primary axillary hyperhidrosis
CM	Chronic migraine
NDO	Neurogenic detrusor overactivity
OAB	Overactive bladder
CP	Cerebral palsy
